# Novel 2-Sulfanylquinazolin-4(3*H*)-one Derivatives as Multi-Kinase Inhibitors and Apoptosis Inducers: A Synthesis, Biological Evaluation, and Molecular Docking Study

**DOI:** 10.3390/molecules28145548

**Published:** 2023-07-20

**Authors:** Ali Altharawi, Mohammed M. Alanazi, Manal A. Alossaimi, Ashwag S. Alanazi, Safar M. Alqahtani, Mohammed H. Geesi, Yassine Riadi

**Affiliations:** 1 Department of Pharmaceutical Chemistry, College of Pharmacy, Prince Sattam Bin Abdulaziz University, Al-Kharj 11942, Saudi Arabia; 2 Department of Pharmaceutical Chemistry, College of Pharmacy, King Saud University, Riyadh 11541, Saudi Arabia; 3 Department of Pharmaceutical Sciences, College of Pharmacy, Princess Nourah Bint Abdulrahman University, Riyadh 84428, Saudi Arabia; 4 Department of Chemistry, College of Science and Humanities in Al-Kharj, Prince Sattam bin Abdulaziz University, Al-Kharj 11942, Saudi Arabia

**Keywords:** quinazolin-4(3*H*)-one, protein kinases, apoptosis, cytotoxicity, molecular docking

## Abstract

The discovery of multi-targeted kinase inhibitors emerged as a potential strategy in the therapy of multi-genic diseases, such as cancer, that cannot be effectively treated by modulating a single biological function or pathway. The current work presents an extension of our effort to design and synthesize a series of new quinazolin-4-one derivatives based on their established anti-cancer activities as inhibitors of multiple protein kinases. The cytotoxicity of the new derivatives was evaluated against a normal human cell line (WI-38) and four cancer lines, including HepG2, MCF-7, MDA-231, and HeLa. The most active compound, **5d**, showed broad-spectrum anti-cancer activities against all tested cell lines (IC_50_ = 1.94–7.1 µM) in comparison to doxorubicin (IC_50_ = 3.18–5.57 µM). Interestingly, compound **5d** exhibited lower toxicity in the normal WI-38 cells (IC_50_ = 40.85 µM) than doxorubicin (IC_50_ = 6.72 µM), indicating a good safety profile. Additionally, the potential of compound **5d** as a multi-targeted kinase inhibitor was examined against different protein kinases, including VEGFR2, EGFR, HER2, and CDK2. In comparison to the corresponding positive controls, compound **5d** exhibited comparable activities in nanomolar ranges against HER2, EGFR, and VEGFR2. However, compound **5d** was the least active against CDK2 (2.097 ± 0.126 µM) when compared to the positive control roscovitine (0.32 ± 0.019 µM). The apoptotic activity investigation in HepG2 cells demonstrated that compound **5d** arrested the cell cycle at the S phase and induced early and late apoptosis. Furthermore, the results demonstrated that the apoptosis pathway was provoked due to an upregulation in the expression of the proapoptotic genes caspase-3, caspase-9, and Bax and the downregulation of the Bcl-2 anti-apoptotic gene. For the in silico docking studies, compound **5d** showed relative binding interactions, including hydrogen, hydrophobic, and halogen bindings, with protein kinases that are similar to the reference inhibitors.

## 1. Introduction

Cancer remains the leading cause of death worldwide, and approximately 19.3 million new cancer cases and 10 million cancer deaths were reported in 2020 [1]. Recently, the American Cancer Society estimated over 1.95 million new cancer cases, and 609,820 cancer deaths are expected to occur in 2023 [2]. The emergence of drug resistance, low selectivity, and high toxicity in the cytotoxic drugs that are currently used have created an urge to discover and develop new classes of anti-cancer agents. When in search of new cancer therapies, many studies have increasingly focused on the development of more potent and selective anti-cancer drugs against pivotal targets, such as protein tyrosine kinases (PTKs) [3,4]. PTKs play a vital role in the control of cascades of biological events that are involved in cell proliferation, differentiation, metastasis, and apoptosis [5,6,7,8]. The upregulation of PTKs drives uncontrolled cell proliferation and often results in the initiation and progression of neoplastic cells. PTKs are mainly classified into two classes according to their structures and locations: receptor tyrosine kinases (RTKs) and non-receptor tyrosine kinases (NRTKs). While NRTKs are cytosolic enzymes, RTKs are transmembrane receptor proteins that transfer transduction signals to downstream effectors such as hormones, growth factors, and polypeptides. Numerous RTKs have been identified and recognized as attractive targets for the further development of novel inhibitors [9]. These receptors mainly include epidermal growth factor receptors (EGFRs), platelet-derived growth factor receptors (PDGFRs), *fibroblast growth factor receptors* (FGFRs), and vascular endothelial growth factor receptors (VEGFRs). For example, the EGFR family, also referred to as HER or ErbB receptors, is composed of EGFR (ErbB1, HER1), HER2 (ErbB2), HER3 (ErbB3), and HER4 (ErbB4). The EGFR family are transmembrane tyrosine kinase receptors that play a significant role in signal transduction pathways [10]. The overexpression of EGFR is known to be involved in various cancers mediated by the stimulation of carcinogenetic processes resulting in cell proliferation, apoptosis, angiogenesis, and invasiveness through main cascades such as Ras/Raf/MAPK and PIK-3/AKT [11,12]. Additionally, the EGFR-signaling cascades are implicated in the activation of the VEGFR, which is recognized as the main process in the stimulation of angiogenesis. Cyclin-dependent kinases (CDKs) are a large family of serine/threonine kinase enzymes that catalyze the phosphorylation of proteins and control the transition of cell cycles through different phases. Likewise, the overexpression of CDKs has been implicated in many cancer types [13]. These kinases have been recognized as valid targets for anti-cancer therapeutics, and up until now, the US FDA has approved more than 73 small molecules regarding protein kinase inhibitors; most are multi-targeted tyrosine kinase inhibitors (MT-TKIs) [14,15,16,17].

The nitrogen-containing heterocycles are well-known and widely investigated bioactive molecules with a broad range of pharmacological activities [18,19]. Quinazoline, for example, is a nitrogen-containing heterocycle for which numerous pharmacological activities have been recognized, including anti-tubercular, anti-malarial, anti-fungal, anti-microbial, anti-viral, and many others [20,21,22,23,24]. Of particular interest, quinazoline-based compounds emerged as privileged scaffolds possessing anti-tumor activity against various cancer cells through the inhibition of different protein kinases [25,26,27]. For instance, gefitinib (Iressa^®^) and erlotinib (Tarceva^®^) are 4-amino quinazoline derivatives that have been shown to exert their anti-cancer activity through the inhibition of epidermal growth factor receptor (EGFR) [28,29]. Idelalisib (Zydelig^®^) is a quinazolin-4(3*H*)-one derivative that acts as an inhibitor of phosphoinositide-3 kinase (PI3K) for the treatment of certain hematological malignancies [30]. Furthermore, some quinazoline-based compounds have also been shown to act as MT-TKIs. For example, lapatinib (Tykreb^®^) is a dual inhibitor that interferes with the HER2/neu and EGFR and is used orally in managing breast cancer and other solid tumors [31]. Vandetanib (Caprelsa^®^) is a 4-anilinoquinazoline derivative and was approved by the FDA in 2011 for the treatment of thyroid cancer. It mainly acts as a potent inhibitor of VEGFR and EGFR [32]. Other quinazoline-based derivatives bearing various moieties, such as benzenesulfonamide, piperazine, and 1,2,4-triazolestyryl, were designed and synthesized as potential MT-TKIs and have shown enhanced in vitro cytotoxic activities against various cancer cell lines [33,34,35]. Previously, we reported the potential of new quinazolinone-based derivatives as a dual inhibitor of VEGFR2 and EGFR [36,37]. 

In the current work, we have extended our effort to synthesize effective quinazolin-4(3*H*)-one derivatives as anti-cancer agents and investigated their potential as MT-TKIs and apoptosis inducers. Novel 2-sulfanylquinazolin-4(3*H*)-one derivatives were synthesized and evaluated for their cytotoxic activities against some human cancer cell lines in addition to normal human cells. Additionally, the potential of the most cytotoxic derivative as a protein kinase inhibitor of EGFR, VEGFR2, HER2, or CDK2, as well as being an apoptosis inducer, was examined. Finally, we have studied the binding affinity of the most active compound to EGFR, VEGFR2, and CDK2 protein kinases through the in silico molecular docking approach.

## 2. Results and Discussion

### 2.1. Chemistry

**Scheme 1:**
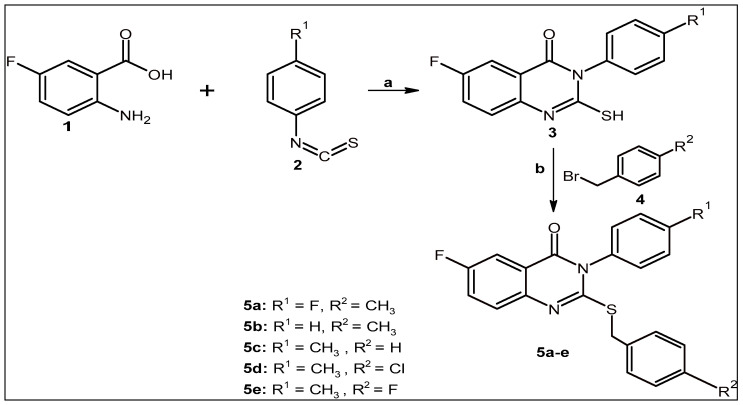
Synthesis route of 2-sulfanylquinazolin-4(3*H*)-one derivatives (**5a**–**e**). Reagents and conditions: (a) EtOH and Et3N, reflux for 1.5 h. (b) Appropriate benzyl bromide (**4**), acetone, and K_2_CO_3_, stir for 10 h.

The procedure employed to prepare the new 2-sulfanylquinazolin-4(3*H*)-one derivatives is described in our previous work [38]. A schematic illustration of the chemical reaction is shown in Scheme 1. In brief, arylisothiocyanate **2a**–**c** (1 mmol) was added dropwise under stirring to a mixture of 5-fluoroanthranilic acid **1** (1 mmol) in absolute ethanol (20 mL), followed by the addition of triethylamine (1.1 mmol, 0.11 g). This mixture was refluxed for 1.5 h to ensure the total consumption of the starting reactants, as determined by thin-layer chromatography (TLC). The mixture was filtered, and the solvent was removed in vacuo. The resulting crude solid was recrystallized from ethanol to achieve the pure product **3a**–**c**. The following step involved reacting intermediates **3a**–**c** (1 mmol) with different benzyl bromides (1 mmol, **4**) in 10 mL of acetone containing anhydrous potassium carbonate (1.5 mmol, K_2_CO_3_) and stirring for 10 h. After the completion of the reaction, the mixture was filtered, and the solvent was removed in vacuo. The resulting solid was recrystallized from ethanol to give the final compounds **5a**–**e** (Supplementary Materials Figures S3–S12).

### 2.2. Biological Evaluation

#### 2.2.1. In Vitro Anti-Proliferative Activity

The in vitro cytotoxic activity of the 2-sulfanylquinazolin-4(3*H*)-one derivatives were evaluated against four human cancer cell lines, namely, hepatocellular carcinoma (HepG2), breast cancer (MCF-7), triple-negative breast cancer (MDA-MB-231), and cervical cancer (HeLa). In addition, the safety profile of the 2-sulfanylquinazolin-4(3*H*)-one derivatives was evaluated against normal human cells (WI38) as a positive control. The standard MTT (3-(4,5-dimethylthiazol-2-yl)-2,5 diphenyl tetrazolium bromide) colorimetric assay protocol was used, as described by Mosmann [39]. Doxorubicin was used as a non-selective reference cytotoxic drug, whereas sunitinib was used as a more selective anti-cancer agent. The results were expressed as growth inhibitory concentration (IC_50_) values. In comparison to doxorubicin and sunitinib, the evaluated compounds showed a different range of anti-proliferative activities against the tested cancer cell lines, as summarized in Table 1. In general, compound **5d,** substituted with 4-chlorophenyl at position 3, was the most cytotoxic derivative against the HepG2, MCF-7, MDA-231, and HeLa cell lines with an IC_50_ of 7.10, 2.48, 1.94, and 6.38, respectively. The cytotoxicity of compound **5d** was comparable to the IC_50_ of doxorubicin in all tested cancer cell lines. Interestingly, compound **5d** displayed less cytotoxic activity on the WI38 cells (IC_50_ = 64.29 µM) when compared to doxorubicin (IC_50_ = 6.72 µM). When compared to sunitinib, compound **5d** showed more potency toward all the tested cell lines with a relatively similar safety profile against the WI38 cell line. Derivatives **5a**–**c** showed less cytotoxic activities when compared to doxorubicin and sunitinib on all tested cancer cell lines, with IC_50_ values ranging from 19 to >100 µM, while **5e** exhibited moderate to strong cytotoxicity (IC_50_ = 19.18 to 38.17 µM).

#### 2.2.2. Kinase Inhibitory Activity

To obtain mechanistic insight into the potential of the most active compound (**5d**) against the tested cell lines, we examined the inhibitory activities against multiple TK enzymes, specifically against EGFR, HER2, VEGFR2, and CDK2 by using erlotinib as a positive control for EGFR and HER2, while sorafenib and roscovitine were used as reference standards for VEGFR2 and CDK2, respectively. The corresponding kinase inhibitory kit assays were used, and the results are expressed as IC_50_ values (µM), as presented in Table 2. In comparison to the positive controls, it can be noted that compound **5d** exhibited comparable activities in nanomolar ranges against EGFR, HER2, and VEGFR2. However, compound **5d** was less active against CDK2 (2.097 ± 0.126 µM) when compared to the positive control roscovitine (0.32 ± 0.019 µM).

#### 2.2.3. Investigation of Apoptosis

##### Annexin V/PI Staining and Cell Cycle Analysis

**Figure 1:**
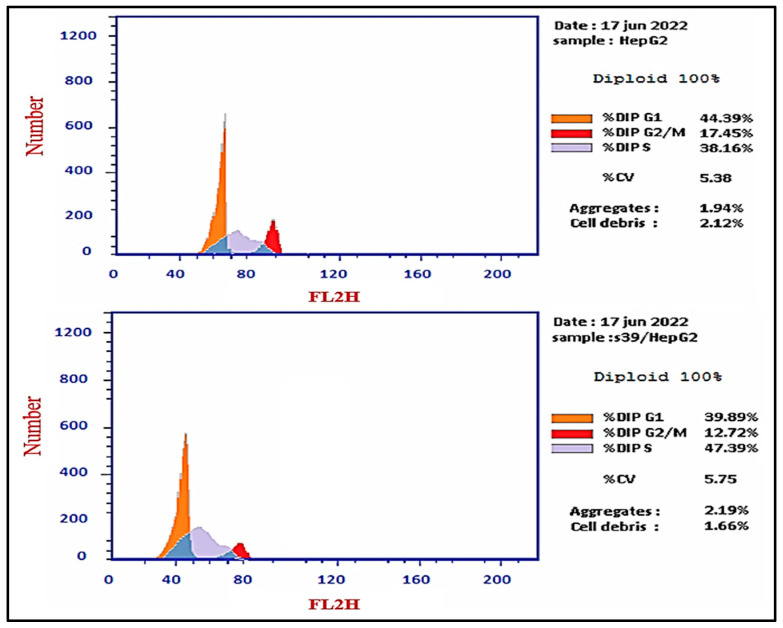

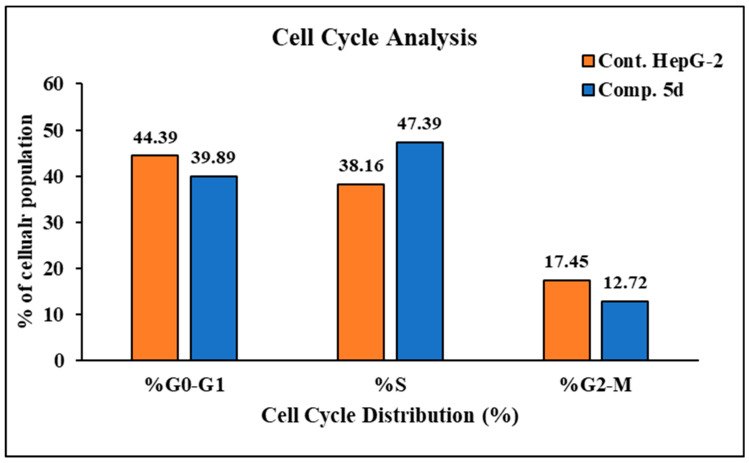
Histograms of DNA content-flow cytometry of untreated (**upper panel**) and treated HepG2 cells (**down panel**) with compound **5d** (IC_50_ values = 7.1 µM; 24 h) and bar representation of the percentage of cell population at each cell cycle.

**Figure 2:**
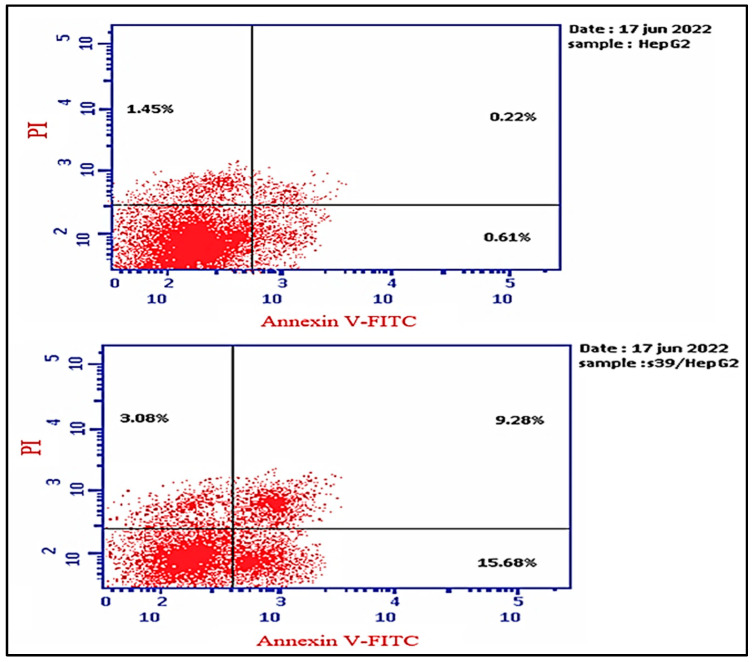

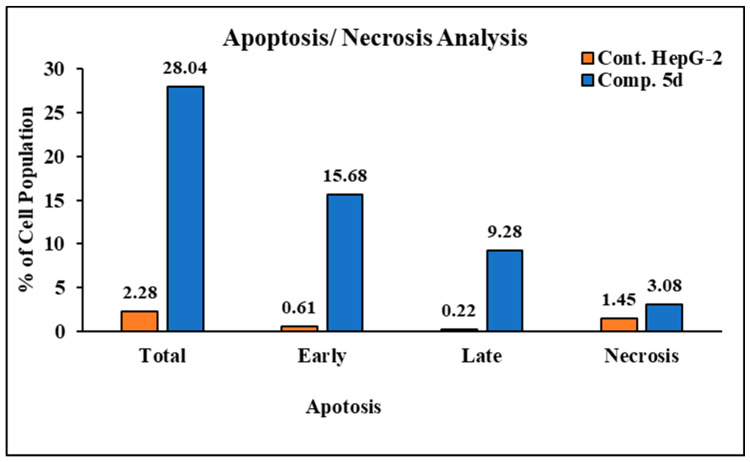
Cryptographs of annexin-V/propidium iodide staining of untreated (**upper panel**) and treated HepG2 cells (**down panel**) with compound **5d** (IC_50_ values = 7.1 µM; 24 h). Q1 (necrotic, AV_−_/PI_+_), Q2 (late apoptotic cells, AV_+_/PI_+_), Q3 (normal cells, AV_−_/PI_−_), Q4 (early apoptosis, AV_+_/PI_−_), and bar representation of the percentage of total apoptosis and cell population in the early and late apoptosis and necrosis.

In order to investigate apoptosis, the most cytotoxic compound in anti-proliferative assay **5d** was further exposed to cell cycle analysis using the standard Annexin V/PI double staining procedure [40,41]. The HepG2 cells were exposed to compound **5d** at a concentration equal to its IC_50_ values (7.1 µM) for 24 h, and the definite phase at which cell cycle arrest took place was determined. The results of the cell cycle analysis are presented in Figure 1. The exposure of HepG2 cells to compound **5d** resulted in a drop in the cell population at the G0-G1 and G2/M phases: 4.5% and 4.73%, respectively. Moreover, an increase in the cell population of 9.23% was observed in the S phase in comparison to the control. Additionally, the results showed that compound **5d** remarkably induced apoptosis at all stages in comparison to the untreated HepG2 cells, where the total apoptosis was enhanced by (28.04%) over the control (2.28%), as shown in Figure 2. Additionally, compound **5d** induced early and late apoptosis as well as necrotic cell death by 7.51%, 3.45%, and 3.08%, respectively, when compared with the untreated HepG2 cells (0.61%, 0.22%, and 1.45%, respectively), as illustrated in Figure 2. The obtained results show that compound **5d** arrested HepG2 cell growth in the S phase and induced early and late apoptosis. 

##### Real-Time Polymerase Chain Reaction (RT-PCR) for the Selected Genes

**Figure 3:**
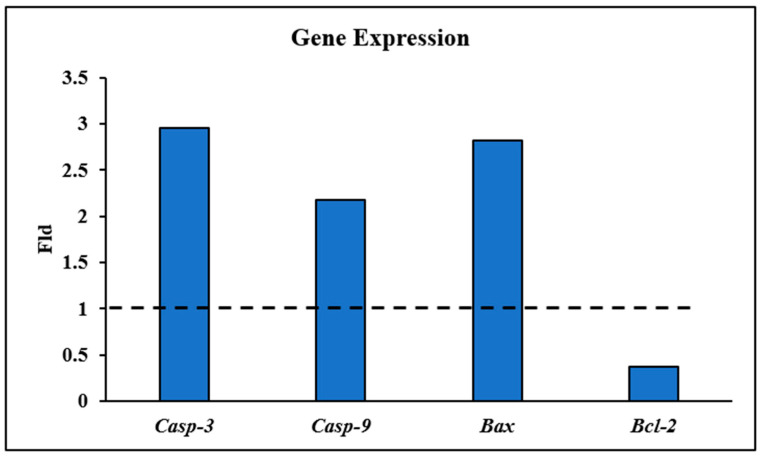
Gene expression representation of Casp-3, Casp-9, and Bax proapoptotic genes and Bcl-2 anti-apoptotic gene in HepG2 cells treated with compound **5d**. The dashed horizontal line represents the control (fold change = 1).

RT-PCR reactions were carried out to investigate the relative gene expression of apoptosis-related genes, including the proapoptotic genes Bax, caspases 3 and 9, and the anti-apoptotic Bcl2 gene [42]. As shown in Figure 3, the apoptotic pathway of the HepG2 cells treated with compound **5d** approximately increased the gene expression of Bax and caspase-3 three-fold and caspase-9 two-fold. On the other hand, it downregulated the expression of the anti-apoptotic gene Bcl2 by 0.4-fold. Therefore, it can be concluded that the induction of apoptosis in HepG2 cells upon treatment with **5d** is associated with the upregulation of proapoptotic genes and the downregulation of anti-apoptotic genes.

### 2.3. Molecular Docking

**Figure 4:**
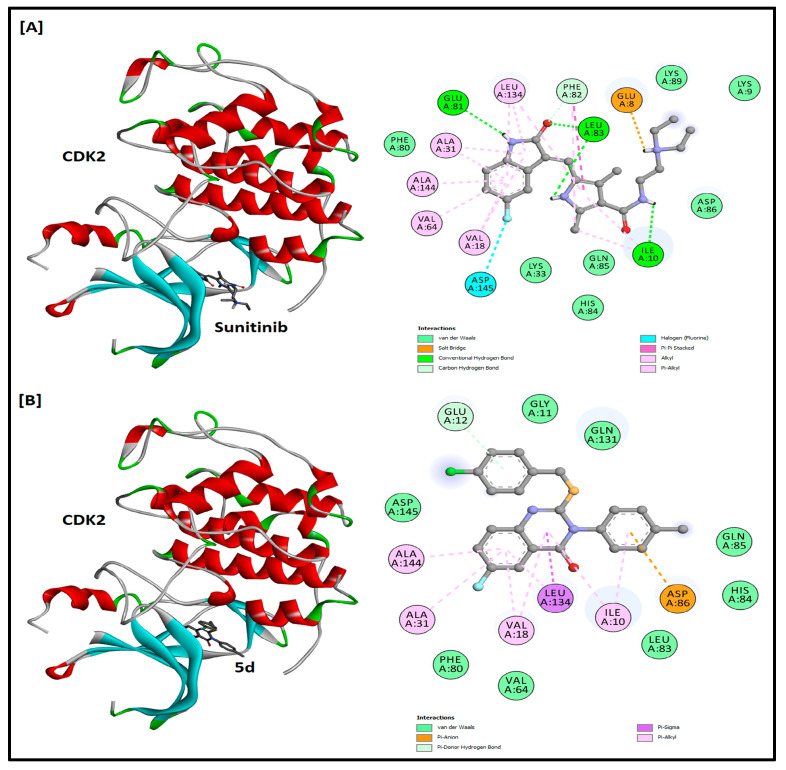
The 2D and 3D docking analysis of sunitinib (**A**) and compound **5d** (**B**) inside the ATP binding pocket of CDK2 protein kinase (PDB ID: 3TI1).

**Figure 5:**
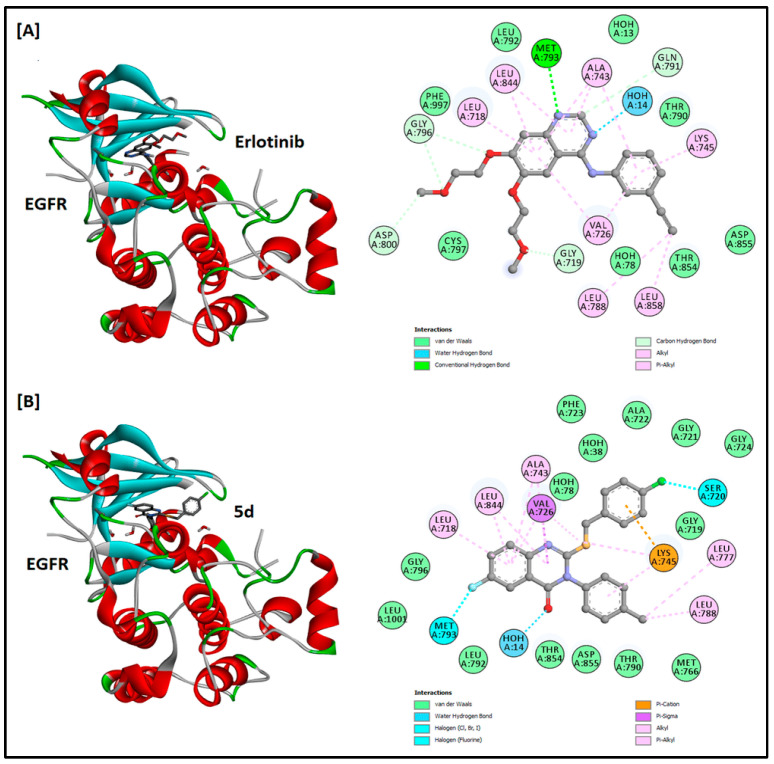
The 2D and 3D docking analysis of erlotinib (**A**) and compound **5d** (**B**) inside the ATP binding pocket of EGFR protein kinase (PDB ID: 3POZ).

**Figure 6:**
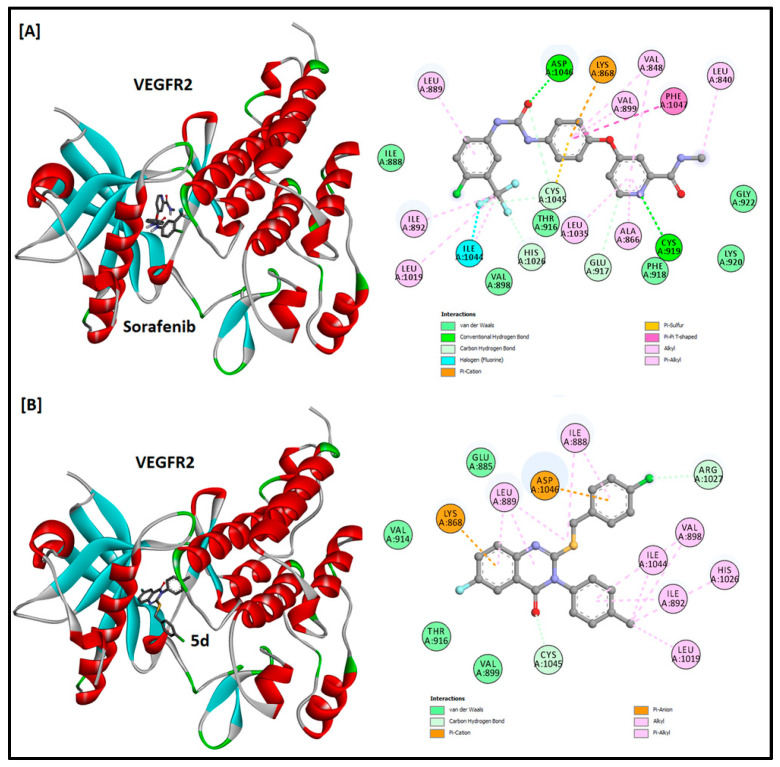
The 2D and 3D docking analysis of sorafenib (**A**) and compound **5d** (**B**) inside the ATP binding pocket of VEGFR2 protein kinase (PDB ID: 3U6J).

Compound **5d** was docked into the ATP binding pocket of CDK2, EFGR, and VEGFR2 to find its potential as an inhibitor of these protein kinases. Sunitinib, erlotinib, and sorafenib were kept as positive controls for CDK2, EFGR, and VEGFR2, respectively. The docking results and the 2D and 3D docking analysis of the positive controls and compound **5d** are summarized in Table 3 and Figure 4, Figure 5 and Figure 6. The binding of sunitinib in the active site formed four hydrogen bonds (one with Glu81, two with Leu83, and one with Ile10). A salt bridge was also observed between Glu8 and the terminal amine of sunitinib, and a halogen bond with Asp145 was favored in the binding as well. In addition, sunitinib was stabilized by several hydrophobic interactions with Val18, Ala31, Val64, Phe82, Leu134, and Ala144. The docking score and binding energy for sunitinib were −9.576 and −81.386 kcal/mol, respectively. The docking score obtained for compound **5d** was −6.786 kcal/mol, while the binding energy was −64.058 kcal/mol. Compound **5d** was found to be stabilized in the active site mainly by hydrophobic interactions with amino acids Ile10, Glu12, Val18, Ala31, Val64, Phe80, Asp 86, Leu134, and Ala144. The docking results of erlotinib were observed in accordance with the literature, as it binds in the active site with two hydrogen bonds, one with Met793 and the other with Thr854, mediated by a water molecule [17]. In a similar pattern, compound **5d** formed a hydrogen bond with Thr854 mediated by a water molecule. The respective docking score and binding energy of erlotinib were −9.660 and −83.352 kcal/mol, while compound **5d** has a docking score of −8.514 and binding energy of −68.117 kcal/mol. Compound **5d** was also found to be stabilized (with two more halogen bonds) with Met793 and Ser720, as well as hydrophobic interactions in the active site. Regarding VEGFR2, sorafenib binds in the active site with two hydrogen bonds with Cys919 and Asp1046 and one halogen bond with Ile1044 and has a docking score of −9.529 and binding energy of −84.199 kcal/mol. Among the three docked compounds, compound 2 showed good affinity, with a docking score of −7.490, a binding energy of −67.081 kcal/mol, and one hydrogen bond with Asp1046. No hydrogen bonds were observed for compound **5d**. The docking score and binding energy for compound **5d** were −6.150 and −46.976 kcal/mol, respectively. Amino acids Leu840, Val848, Ala866, Lys868, Leu889, Ile892, Val899, Leu1019, His1026, Leu1035, Cys1045, and Phe1047 were found to be involved in hydrophobic interactions within the VEGFR2 active site.

## 3. Materials and Methods

### 3.1. Synthesis 

First, arylisothiocyanate **2a**–**c** (1 mmol) was reacted under stirring with a mixture of 5-fluoroanthranilic acid **1** (1 mmol) in absolute ethanol (20 mL), and then triethylamine (1.1 mmol, 0.11 g) was added. This mixture was refluxed for 1.5 h to ensure the total consumption of the starting reactants, as determined by thin-layer chromatography (TLC). The mixture was filtered, and the solvent was removed in vacuo. The resulting crude solid was recrystallized from ethanol to achieve the pure product **3a**–**c**. The following step involved reacting intermediates **3a**–**c** (1 mmol) with different benzyl bromides (1 mmol, **4**) in 10 mL of acetone containing anhydrous potassium carbonate (1.5 mmol, K_2_CO_3_) and stirring for 10 h. After the completion of the reaction, the mixture was filtered, and the solvent was removed in vacuo. The resulting solid was recrystallized from ethanol to give the final compounds **5a**–**e**.

The melting points of the prepared derivatives were determined in open capillaries using melting point apparatus (Sanyo Gallenkamp, South Borough, UK). Pre-coated silica gel thin-layer chromatography (TLC) plates (silica gel 0.25 mm, 60G F254. Merck, Germany) were employed for the TLC experiment. A developing solvent system consisting of a mixture of (*n*-hexane: EtOAc (80:20 *v*/*v*)) was used as a mobile phase, and the spots were recorded using ultraviolet light. Elucidation of the total proton and environment was made by recording ^1^H NMR spectra using the NMR instrument BRUCKER-PLUS (500 MHz), while ^13^C NMR spectra were run at 125 MHz. CDCl_3_ was used as a solvent for all compounds except 5a, where DMSO was used as the solvent. The chemical shifts are expressed in δ-values (ppm) relative to TMS as an internal standard. All chemicals and solvents were purchased from Sigma Aldrich and AK Scientific. 

Intermediate (**3a**) white solid (89%); M.P.: 142–144 °C; IR (cm^−1^): 1562 (C = C), 1681 (C = O), 3046 (ArC-H); ^1^H NMR (500 MHz, CDCl_3_) δ: 2.48 (s, 3H, CH_3_), 7.46 (d, 2H, H_Ar_, *J* = 7.5 Hz), 7.55 (d, 1H, H_8_, *J* = 10 Hz), 7.62 (d, 1H, H_7_, *J* = 10 Hz), 8.01 (s, 1H, H_5_), 8.2 (d, 2H, H_Ar_, *J* = 7.5 Hz). ^13^C NMR (125 MHz, CDCl_3_) δ: 21.71 (CH_3_), 116.99 (CH), 127.93 (CH), 128.62 (CH), 129.18 (Cq), 129.41 (CH × 2), 129.80 (CH × 2), 138.25 (Cq), 139.15 (Cq), 139.33 (Cq), 144.96 (Cq), 155.85 (Cq), 159.84 (Cq); MS: *m*/*z* 286.

**3-(4-fluoro-Arenyl)-6-fluoro-2-(4-methyl-benzylsulfanyl)-3*H*-quinazolin-4-one (5a)** white solid (82%); MP: 166−168 °C; IR (cm^−1^): 1560 (C = C), 1689 (C = O), 3042 (ArC-H); ^1^H NMR (500 MHz, DMSO) δ: 2.44 (s, 3H, CH_3_), 4.39 (s, 2H, SCH_2_), 7.09–7.13 (m, 2H, H_Ar_, *J* = 10 Hz), 7.36–7.40 (m, 2H, H_Ar,_ *J* = 10 Hz), 7.47–7.53 (m, 4H, H_Ar_, *J* = 10 Hz), 7.61 (d, 1H, H_8_), 7.69 (m, 1H, H_7_), 7.88 (s, 1H, H_5_). ^13^C NMR (125 MHz, CDCl_3_) δ: 21.32 (CH_3_), 36.72 (CH_2_), 117.02 (CH), 117.20 (CH), 118.75 (CH × 2), 119.60 (CH), 124.98 (Cq), 127.32 (CH), 129.63 (CH), 129.77 (CH × 3), 131.07 (Cq), 132.78 (CH), 136.96 (Cq), 142.36 (Cq), 144.80 (Cq), 157.32 (Cq), 161.25 (Cq), 167.08 (Cq), 196.96 (Cq); MS: *m*/*z* 394.7.

**6-Fluoro-2-(4-methyl-benzylsulfanyl)-3-Arenyl-3*H*-quinazolin-4-one (5b)** white solid (86%); M.P.: 158-160 °C; IR (cm^−1^): 1572 (C = C), 1692 (C = O), 3036 (ArC-H); ^1^H NMR (500 MHz, CDCl_3_) δ: 2.51 (s, 3H, CH_3_), 4.47 (s, 2H, SCH_2_), 7.20–7.24 (m, 2H, H_Ar_, *J* = 10 Hz), 7.27–7.34 (m, 5H, H_Ar_, *J* = 10 Hz), 7.42–7.44 (d, 2H, H_Ar_, *J* = 10 Hz), 7.59–7.66 (m, 2H, H_7_, H_8_, *J* = 10 Hz), 8.08 (s, 1H, H_5_). ^13^C NMR (125 MHz, CDCl_3_) δ: 21.36 (CH_3_), 37.17 (CH_2_), 116.73 (CH), 116.92 (CH), 119.60 (CH), 126.18 (CH), 126.73 (CH), 127.62 (CH), 128.66 (CH × 2), 129.46 (CH × 2), 131.30 (CH), 131.83 (Cq), 136.14 (CH), 136.27 (Cq), 136.42 (Cq), 145.89 (Cq), 155.91 (Cq), 161.95 (Cq), 162.27 (Cq), 164.26 (Cq); MS: *m*/*z* 376.7.

**2-Benzylsulfanyl-6-fluoro-3-(4-methyl-Arenyl)-3*H*-quinazolin-4-one (5c)** white solid (81%); M.P.: 156-158 °C; IR (cm^−1^): 1578 (C = C), 1678 (C = O), 3056 (ArC-H); ^1^H NMR (500 MHz, CDCl_3_) δ: 2.49 (s, 3H, CH_3_), 4.45 (s, 2H, SCH_2_), 7.16–7.26 (m, 2H, H_Ar_, *J* = 5 Hz), 7.27–7.33 (m, 5H, H_Ar_, *J* = 10 Hz), 7.41–7.43 (d, 2H, H_Ar_, *J* = 10 Hz), 7.58–7.63 (m, 2H, H_7_, H_8_, *J* = 10 Hz), 8.06 (s, 1H, H_5_). ^13^C NMR (125 MHz, CDCl_3_) δ: 21.52 (CH_3_), 37.33 (CH_2_), 116.89 (CH), 117.08 (CH), 119.76 (CH), 126.34 (CH), 126.89 (CH), 127.78 (CH), 128.82 (CH), 129.62 (CH), 131.39 (CH), 131.46 (Cq), 131.96 (CH), 131.99 (CH), 136.30 (CH), 136.43 (Cq), 136.58 (Cq),145.05 (Cq), 156.07 (Cq), 162.11 (Cq), 162.43 (Cq), 164.42 (Cq); MS: *m*/*z* 376.7.

**2-(4-Chloro-benzylsulfanyl)-6-fluoro-3-(4-methylArenyl)-3*H*-quinazolin-4-one (5d)** white solid (78%); M.P.: 164-166 °C; IR (cm^−1^): 1562 (C = C), 1684 (C = O), 3061 (ArC-H); ^1^H NMR (500 MHz, CDCl_3_) δ: 2.34 (s, 3H, CH_3_), 4.28 (s, 2H, SCH_2_), 6.99–7.03 (m, 2H, H_Ar_, *J* = 10 Hz), 7.26–7.30 (m, 2H, H_Ar,_ *J* = 10 Hz), 7.37–7.42 (m, 4H, H_Ar_, *J* = 10 Hz), 7.50–7.52 (d, 1H, H_8_, *J* = 10 Hz), 7.57–7.59 (m, 1H, H_7_), 7.79 (d, 1H, H_5_, *J* = 5 Hz). ^13^C NMR (125 MHz, CDCl_3_) δ: 21.33 (CH_3_), 36.74 (CH_2_), 117.02 (CH), 117.21 (CH), 118.76 (CH × 2), 119.61 (CH), 124.99 (CH), 127.33 (CH), 129.64 (CH), 129.78 (CH × 3), 131.08 (Cq), 132.79 (Cq), 136.97 (Cq), 142.37 (Cq), 144.81 (Cq), 157.33 (Cq), 161.25 (Cq), 167.09 (Cq), 196.97 (Cq); MS: *m*/*z* 410.8.

**2-(4-Fluoro-benzylsulfanyl)-6-fluoro-3-(4-methylArenyl)-3*H*-quinazolin-4-one (5e)** white solid (73%); M.P.: 170-172 °C; IR (cm^−1^): 1548 (C = C), 1669 (C = O), 3058 (ArC-H); ^1^H NMR (500 MHz, CDCl_3_) δ: 2.33 (s, 3H, CH_3_), 4.28 (s, 2H, SCH_2_), 6.98–7.02 (m, 2H, H_Ar_, *J* = 10 Hz), 7.25–7.29 (m, 2H, H_Ar,_ *J* = 10 Hz), 7.36–7.42 (m, 4H, H_Ar,_ *J* = 10 Hz), 7.49–7.51 (d, 1H, H_8_, *J* = 10 Hz), 7.56 (m, 1H, H_7_, *J* = 10 Hz), 7.77 (d, 1H, H_5_, *J* = 5 Hz). ^13^CNMR (125 MHz, CDCl_3_) δ: 20.77 (CH_3_), 36.18 (CH_2_), 116.46 (CH), 116.65 (CH), 118.20 (CH × 2), 119.05 (CH), 124.43 (CH), 126.77 (CH), 129.08 (CH × 2), 129.22 (Cq), 130.52 (CH), 132.22 (Cq), 136.41 (Cq), 141.81 (Cq), 144.25 (Cq), 156.77 (Cq), 160.69 (Cq), 166.53 (Cq), 196.41 (Cq); MS: *m*/*z* 394.7.

### 3.2. Biological Testing

#### 3.2.1. *In Vitro* Anti-Proliferative Activity 

A standard MTT assay protocol was employed to assess the anti-proliferative activity of the synthesized compounds against normal human cells (WI38) and four human cancer cell lines, namely, hepatocellular carcinoma (HepG2), breast cancer (MCF-7), triple-negative breast cancer (MDA-MB-231), and cervical cancer (HeLa). The cell lines were obtained from ATCC (American Type Culture Collection). The anti-cancer activity was measured quantitatively using an MTT assay protocol as follows: Cell lines were cultured in RPMI-1640 medium with 10% fetal bovine serum and 1% antibiotics (100 units/mL penicillin and100 µg/mL streptomycin) at 37 °C in a 5% CO_2_ incubator. Cell lines were seeded at a density of 1.0 × 10^4^ cells/well in a 96-well plate and incubated for 48 h at 37 °C under 5% CO_2_. After incubation, the cells were treated with different concentrations of the synthesized compounds and incubated for 24 h. Afterward, the cells were washed with PBS, and 20 µL of MTT solution at 5 mg/mL was added, all incubated for 4 h. The MTT solution was discarded, and 100 µL of dimethyl sulfoxide (DMSO) was added per well. The dissolved purple formazan products were measured and recorded at an absorbance of 570 nm using a plate reader (EXL 800, USA). The relative cell viability in percentage was calculated as (A_570_ of treated cells/A_570_ of untreated cells) × 100. 

#### 3.2.2. *In Vitro* VEGFR2 Kinase Assay 

The inhibitory activity of compound **5d** against CDK2, HER2, EGFR, and VEGFR2 was evaluated using the corresponding human kinases ELISA kit (Enzyme-Linked Immunosorbent Assay). In brief, specific antibodies for each kinase were seeded on a 96-well plate, and 100 μL of the standard solution or compound **5d** was added. Afterward, the plate was incubated at room temperature for 2.5 h and then washed with PBS. A total of 100 μL of the prepared biotin antibody was added and incubated at room temperature for an additional 1 h, and then 100 μL of streptavidin solution was added and incubated at room temperature for another 45 min. After washing, 100 μL of TMB substrate reagent was added and incubated for 30 min at room temperature. Finally, about 50 μL of the stop solution was added and then read at 450 nm immediately. A standard curve was drawn, with concentrations on the X-axis and absorbance on the Y-axis. 

#### 3.2.3. Flow Cytometry Analysis of Cell Cycle 

Flow cytometry analysis was conducted to assess the effect of compound **5d** on the cell cycle distribution in HepG2 cells. The Flow Cytometry Kit for cell cycle analysis (ab139418_Propidium Iodide Flow Cytometry Kit/BD) was used in this test. First, the HepG2 cells were seeded in six-well plates at a density of 2 × 10^5^ cells/well and incubated for 24 h. In the second step, 10% FBS was added, and the cells were incubated at 37 °C and 5% CO_2_. The medium was replaced with (DMSO 1% *v*/*v*) containing 7.1 µM of compound **5d** and then incubated for 24 h. After that, the cells were fixed with 70% ethanol at 4 °C for 12 h and washed with cold PBS, and then rinsed with PBS. In the last step, 100 μL of RNase A was added to each well and incubated at 37 °C for 30 min and stained with 400 μL PI in the dark at room temperature for an additional 30 min. The stained cells were measured using an Epics XL-MCL™ Flow Cytometer (Beckman Coulter), and the data were analyzed using Flowing software (version 2.5.1, Turku Centre for Biotechnology, Turku, Finland). 

#### 3.2.4. Annexin V-FITC Apoptosis Assay 

The apoptotic and necrotic effect of compound **5d** was investigated using flow cytometry, as described in [43]. The HepG2 cells were treated with 7.1 µM of compound **5d** for 24 h. After treatment, the cells were collected by trypsinization, centrifuged, and washed two times with PBS. A suspension of cells in 500 μL binding buffer was double stained with 5 μL Annexin V-FITC and 5 μL PI in the dark at room temperature for 15 min. The stained cells were measured using an Epics XL-MCL™ Flow Cytometer (Beckman Coulter), and the data were analyzed using Flowing software (version 2.5.1, Turku Centre for Biotechnology, Turku, Finland).

#### 3.2.5. RT-PCR of Selected Apoptosis-Related Genes

The level of apoptotic markers caspase-3, caspase-9, Bax, and Bcl-2 was measured using BIORAD iScript TM One-Step RT-PCR kit with SYBR^®^ Green. According to the manufacturer’s instructions, the kit was used in three subsequent steps.

1. RNA isolation and reverse transcription.

mRNA isolation of up to 1 × 10^7^ cells was carried out using an RNeasy extraction kit. Cells were disrupted in RNeasy Lysis Buffer (RLT buffer) and homogenized. Ethanol was then added to make a lysate and provoke the selective binding of RNA to the RNeasy membrane. The sample was then applied to the RNeasy mini spin column, where total RNA binds to the membrane while contaminants pass through. The RNA was then eluted in RNase-free water.

2. Master mix preparation.

All the following reagents were mixed to make about 50 μL mixture: 2X SYBR^®^ Green RT-PCR reaction mixture (25 μL), forward primer (10 μM; 1.5 μL), reverse primer (10 μM; 1.5 μL), nuclease-free H2O (11 μL), RNA template (1 pg to 100 ng total RNA; 10 μL) and iScript reverse transcriptase for One-Step RT-PCR (1 μL).

3. Amplification protocol.

The whole reaction mixture was incubated in a real-time thermal detection system (Rotorgene) as follows: cDNA synthesis (10 min at 50 °C), iScript reverse transcriptase inactivation (5 min at 95 °C), PCR cycling and detection (30–45 cycles):10 s at 95 °C and 30 s at 55 °C to 60 °C (data collection step) and melt curve analysis: 1 min at 95 °C, 1 min at 55 °C and 10 s at 55 °C–95 °C (80 cycles, increasing each by 0.5 °C each cycle).

### 3.3. Molecular Docking 

The 2D structures of compounds **5d**, sunitinib, erlotinib, and sorafenib were drawn on ChemDraw Ultra (ver. 12) and saved in the MDL-SD file format. The 2D structures were prepared for molecular docking studies by the LigPrep program of Maestro (ver. 12.8.117), Schrödinger. The co-crystallized structures of the protein kinases CDK2 (PDB ID: 3TI1, resolution: 1.99 Å), EFGR (PDB ID: 3POZ, resolution: 1.50 Å), and VEGFR2 (PDB ID: 3U6J, resolution: 2.15 Å) were downloaded from the protein data bank in the pdb format [44,45,46]. The crystal structures were prepared by a protein preparation wizard in Maestro. In short, the pdb files were imported and preprocessed. The protein chains other than A were deleted. All the water molecules were deleted, except in the case of EGFR, where the water molecules within 3 Å of the ligand were retained, as they are reported to be essential for the binding of the ligands. Heteroatoms, other than the ligands, were deleted. The protein structures were optimized, and their energy was minimized by the OPLS4 force field with heavy atoms converged to RMSD 0.30 Å. The refined structures were used to generate a glide grid file by selecting the co-crystallized ligands. To validate the docking protocol, the co-crystallized ligands were first docked by glide XP docking into the ATP binding pocket, and the RMSD between the native and docked conformation was calculated. After validation, compounds 1, 2, 3, sunitinib, erlotinib, and sorafenib were docked by glide XP docking [47]. The docking score, glide emodel, and H-bond interactions were noted. The top-ranked poses of the docked ligands were saved in pdb format, and the 2D and 3D interaction poses were reproduced by Discovery Studio Visualizer (ver. 20.1.0.19295). 

## 4. Conclusions

In conclusion, new derivatives of 2-sulfanylquinazolin-4(3*H*)-one derivatives were synthesized, and their in vitro cytotoxic activities against normal human cells (WI38) and some human cancer cell lines (MCF-7, MDA-MB-231, HepG2, and HeLa) were evaluated. In comparison to doxorubicin and sunitinib, the evaluated compounds showed weak-to-strong anti-proliferative activities against the tested cancer cell lines. Among the derivatives, compound **5d** was the most potent derivative and exhibited broad-spectrum cytotoxicity against all cancer cell lines under investigation. Moreover, compound **5d** was the least toxic in the normal human cell line when compared to the other derivatives and doxorubicin. The potential of compound **5d** as a muti-targeted kinase inhibitor was investigated in HepG2 cells. The results revealed inhibitory effects in the nanomolar range against VEGFR2, EGFR, HER2, and CDK2 kinases when compared to the corresponding positive reference. Additionally, the apoptosis induction assessment indicated that compound **5d** was able to arrest the cell cycle at the S phase and induced apoptosis 10-fold when compared to untreated HepG2 cells. The induction of apoptosis from compound **5d** was associated with its effect on the upregulation of caspase-3, caspase-9, and Bax and the downregulation of Bcl-2 expression. The in silico molecular docking studies showed good binding affinity regarding compound **5d** in the ATP binding pocket of the tested kinases when compared to the reference inhibitors. Based on our findings, compound **5d** showed promising multi-kinase inhibition and apoptosis induction, which can serve as a lead for the further development of potent cancer therapeutics.

## Figures and Tables

**Table 1:** Structures and cytotoxicity results of 2-sulfanylquinazolin-4(3*H*)-one derivatives against normal cell line (WI-38) and cancer cell lines (HepG2, MCF-7, MDA-MB-231, and HeLa) using standard MTT assay.

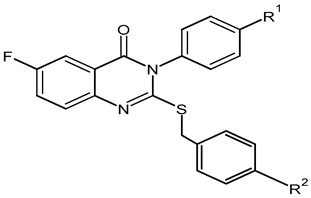							
Comp.	*In Vitro* Cytotoxicity IC_50_ (µM) ^a,b^						
	R^1^	R^2^	WI38	HepG2	MCF-7	MDA-MB-231	HeLa
**3a**	CH_3_	-H	36.54 ± 2.5	82.54 ± 4.3	77.25 ± 4.0	65.48 ± 3.7	73.71 ± 3.9
**5a**	F	-CH_3_	28.70 ± 2.2	>100	86.02 ± 4.4	79.14 ± 4.0	83.23 ± 4.1
**5b**	H	-CH_3_	>100	85.01 ± 4.6	91.18 ± 4.8	78.26 ± 3.9	>100
**5c**	CH_3_	H	40.85 ± 2.6	34.28 ± 2.4	51.88 ± 3.1	26.87 ± 1.9	31.25 ± 2.1
**5d**	CH_3_	Cl	64.29 ± 3.5	7.10 ± 0.6	2.48 ± 0.2	1.94 ± 0.1	6.38 ± 0.4
**5e**	CH_3_	F	55.81 ± 3.2	29.54 ± 2.2	25.20 ± 1.9	19.18 ± 1.5	38.17 ± 2.3
Dox.	----	----	6.72 ± 0.5	4.50 ± 0.2	4.17 ± 0.2	3.18 ± 0.1	5.57 ± 0.4
Sunitinib	----	----	77.33 ± 4.3	11.35 ± 1.0	24.83 ± 1.8	19.04 ± 1.5	21.84 ± 1.7

^a^ IC_50_ values are the mean ± SD of three separate experiments. ^b^ IC_50_ (µM): <10 (potent), 11–50 (moderate), and >50 (weak or negligible).

**Table 2:** Inhibitory activities of compound **5d** against EGFR, HER2, VEGFR2, and CDK2 kinases.

Compound	Kinase Inhibition IC_50_ (µM) ^a^			
	EGFR	HER2	VEGFR2	CDK2
**5d**	0.22 ± 0.013	0.184 ± 0.008	0.336 ± 0.009	2.097 ± 0.126
Positive Control	0.036 ± 0.002 ^b^	0.031 ± 0.002 ^b^	0.042 ± 0.002 ^c^	0.32 ± 0.019 ^d^

^a^ IC_50_ values are the mean ± SD of three separate experiments. ^b^ erlotinib; ^c^ sorafenib; ^d^ roscovitine.

**Table 3:** Glide XP docking of compound **5d** and the respective standard positive controls (sunitinib, erlotinib, and sorafenib) into the ATP binding pocket of CDK2, EFGR, and VEGFR2.

	CDK2 (PDB ID: 3TI1)			EGFR (PDB ID: 3POZ)			VEGFR2 (PDB ID: 3U6J)		
Ligands	Docking Score	Glide Emodel (Kcal/mol)	H-Bond Interactions	Docking Score	Glide Emodel (Kcal/mol)	H-Bond Interactions	Docking Score	Glide Emodel (Kcal/mol)	H-Bond Interactions
Compound **5d**	−6.786	−64.058	---	−8.514	−68.117	-H_2_O-Thr854	−6.150	−46.976	---
Sunitinib	−9.576	−81.386	Leu83, Glu81, Ile10, Glu8	---	---	---	---	---	---
Erlotinib	---	---	---	−9.660	−83.352	Met793, -H_2_O-Thr854	---	---	---
Sorafenib	---	---	---	---	---	---	−9.529	−84.199	Cys919, Asp1046

## Data Availability

Not applicable.

## Supplementary Materials

The following supporting information can be downloaded at: https://www.mdpi.com/article/10.3390/molecules28145548/s1.
