# Supplementary material for: Novel 2-Sulfanylquinazolin-4(3H)-one Derivatives as Multi-Kinase Inhibitors and Apoptosis Inducers: A Synthesis, Biological Evaluation, and Molecular Docking Study
Source: Molecules. 2023 Jul 20;28(14):5548. doi: 10.3390/molecules28145548 (PMC10383864; doi:10.3390/molecules28145548)
Supplement: Supplementary file 1 [file molecules-28-05548-s001.zip › molecules-2455885-supplementary.pdf]

The antiproliferative, multi-kinase inhibition and apoptotic effects of novel 2-sulfanylquinazolin-4(3*H*)-one derivatives: synthesis, biological evaluation, and molecular docking study

Ali Altharawi[1*], Mohammed M. Alanazi[2], Manal A. Alossaimi[1], Ashwag S. Alanazi[3], Safar M. Alqahtani[1], Mohammed H. Geesi[4] and Yassine Riadi[1]

[1] Department of Pharmaceutical Chemistry, College of Pharmacy, Prince Sattam Bin Abdulaziz University, Al-Kharj 11942, Saudi Arabia.

[2] Department of Pharmaceutical Chemistry, College of Pharmacy, King Saud University, Riyadh 11541, Saudi Arabia.

[3] Department of Pharmaceutical Sciences, College of Pharmacy, Princess Nourah Bint Abdulrahman University, Riyadh 84428, Saudi Arabia.

[4] Department of Chemistry, College of Science and Humanities in Al-Kharj, Prince Sattam bin Abdulaziz University, Al-Kharj 11942, Saudi Arabia.

List of Supplementary Information Figures

S1. ^{1}H NMR of intermediate (3a).

S2. ^{13}C NMR of intermediate (3a).

S3. ^{1}H NMR of 3-(4-fluoro-phenyl)-6-fluoro-2-(4-methyl-benzylsulfanyl)-3H-quinazolin-4-one (5a).

S4. ^{13}C NMR of 3-(4-fluoro-phenyl)-6-fluoro-2-(4-methyl-benzylsulfanyl)-3H-quinazolin-4-one (5a).

S5. ^{1}H NMR of 6-Fluoro-2-(4-methyl-benzylsulfanyl)-3-phenyl-3H-quinazolin-4-one (5b).

S6. ^{13}C NMR of 6-Fluoro-2-(4-methyl-benzylsulfanyl)-3-phenyl-3H-quinazolin-4-one (5b).

S7. ^{1}H NMR of 2-Benzylsulfanyl-6-fluoro-3-(4-methyl-phenyl)-3H-quinazolin-4-one (5c).

S8. ^{13}C NMR of 2-Benzylsulfanyl-6-fluoro-3-(4-methyl-phenyl)-3H-quinazolin-4-one (5c).

S9. ^{1}H NMR of 2-(4-Chloro-benzylsulfanyl)-6-fluoro-3-(4-methylphenyl)-3H-quinazolin-4-one (5d).

S10. ^{13}C NMR of 2-(4-Chloro-benzylsulfanyl)-6-fluoro-3-(4-methylphenyl)-3H-quinazolin-4-one (5d).

S11. ^{1}H NMR of 2-(4-Fluoro-benzylsulfanyl)-6-fluoro-3-(4-methylphenyl)-3H-quinazolin-4-one (5e).

S12. ^{13}C NMR of 2-(4-Fluoro-benzylsulfanyl)-6-fluoro-3-(4-methylphenyl)-3H-quinazolin-4-one (5e).

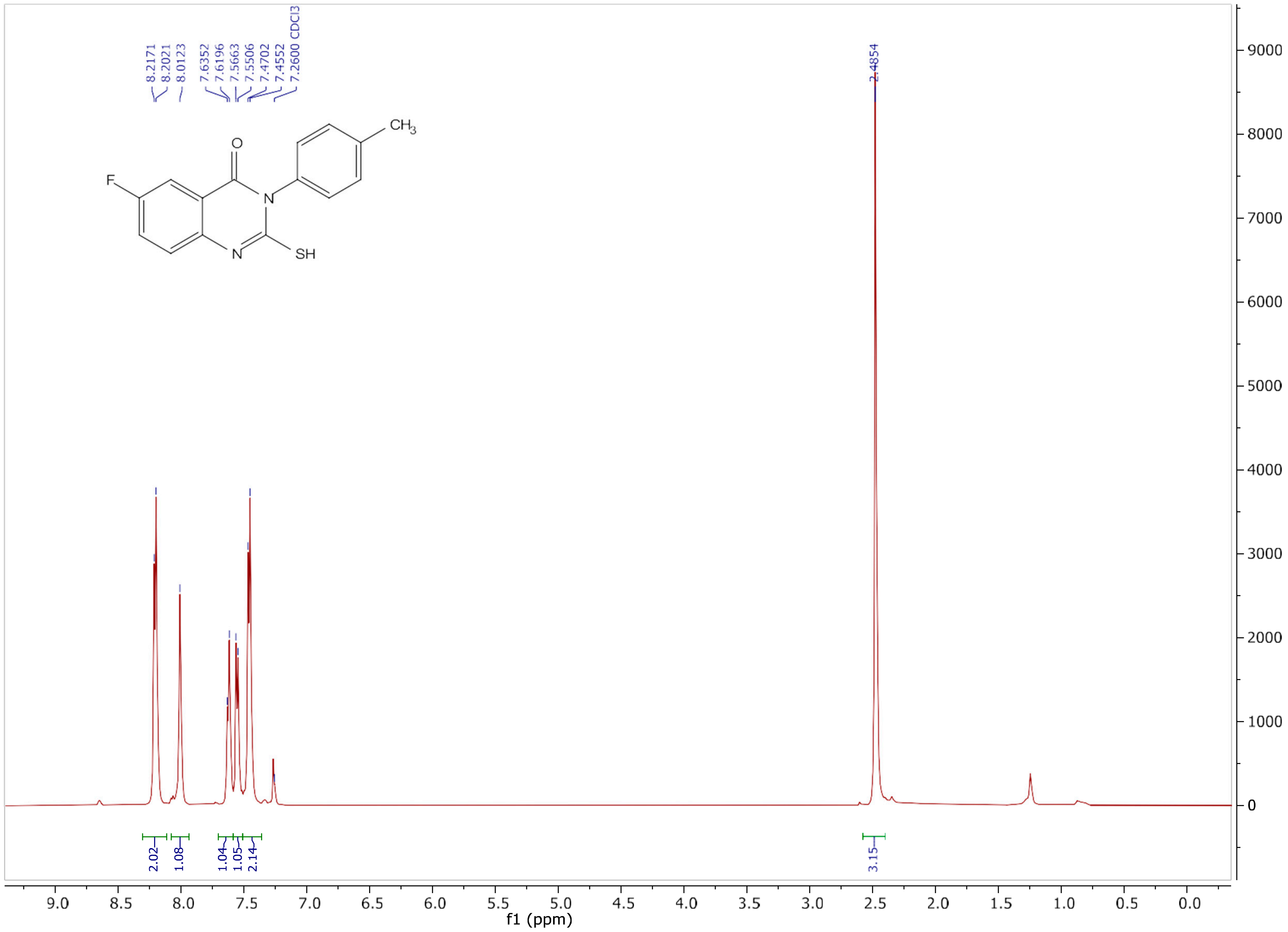

Figure S1. ^{1}H NMR of intermediate **3a**.

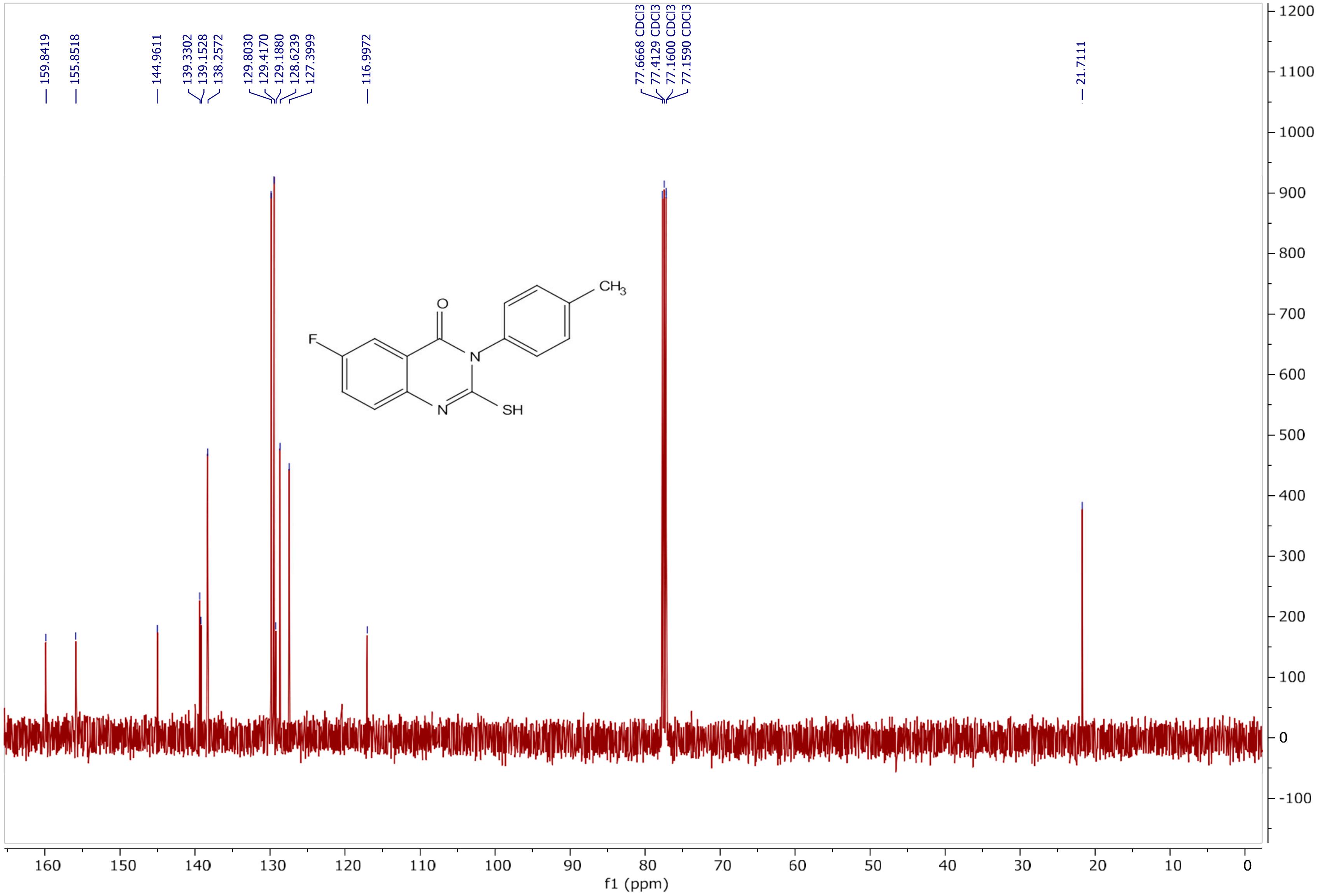

Figure S2. ^{13}C NMR of intermediate **3a**.

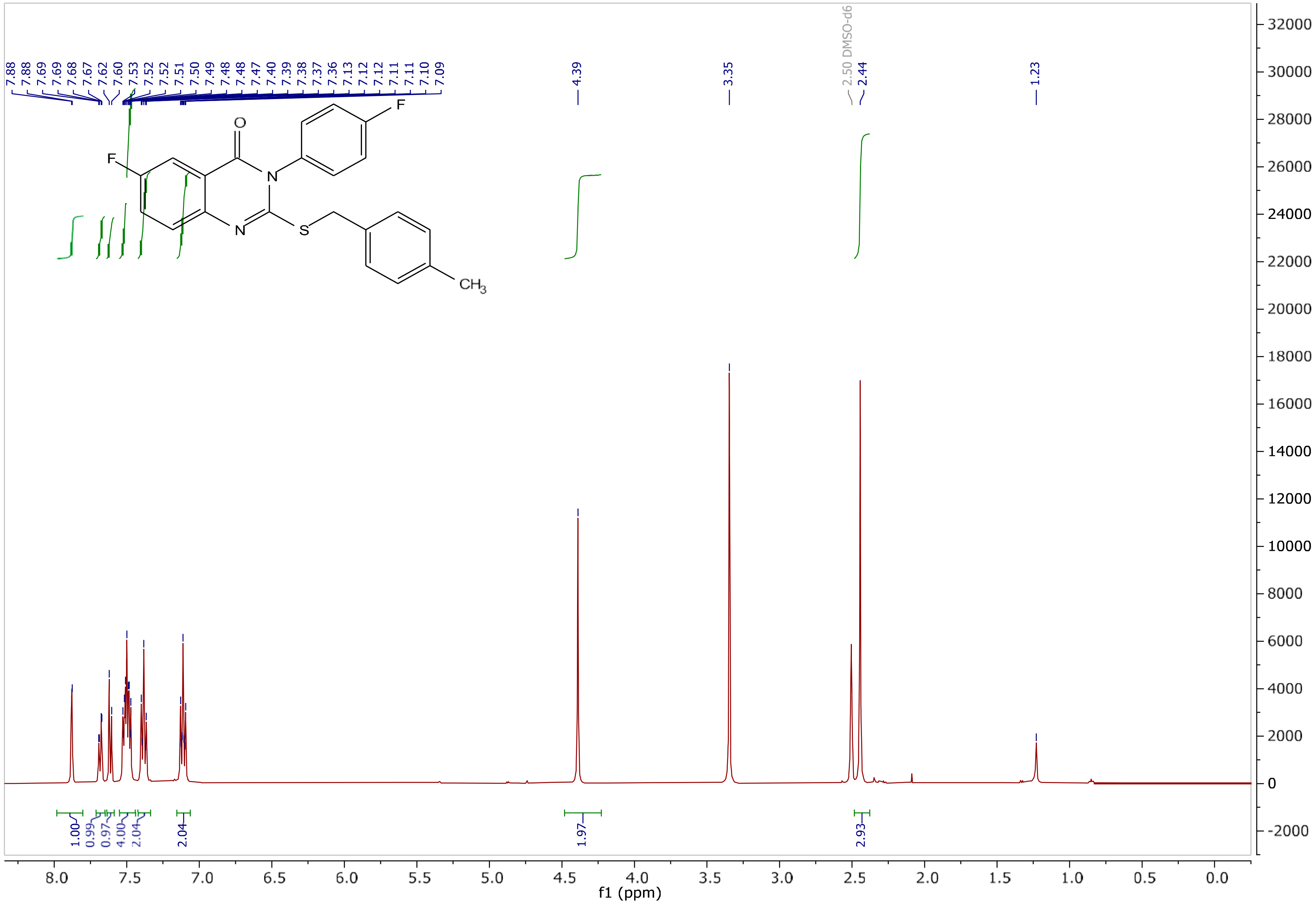

Figure S3. ^{1}H NMR of intermediate **5a**.

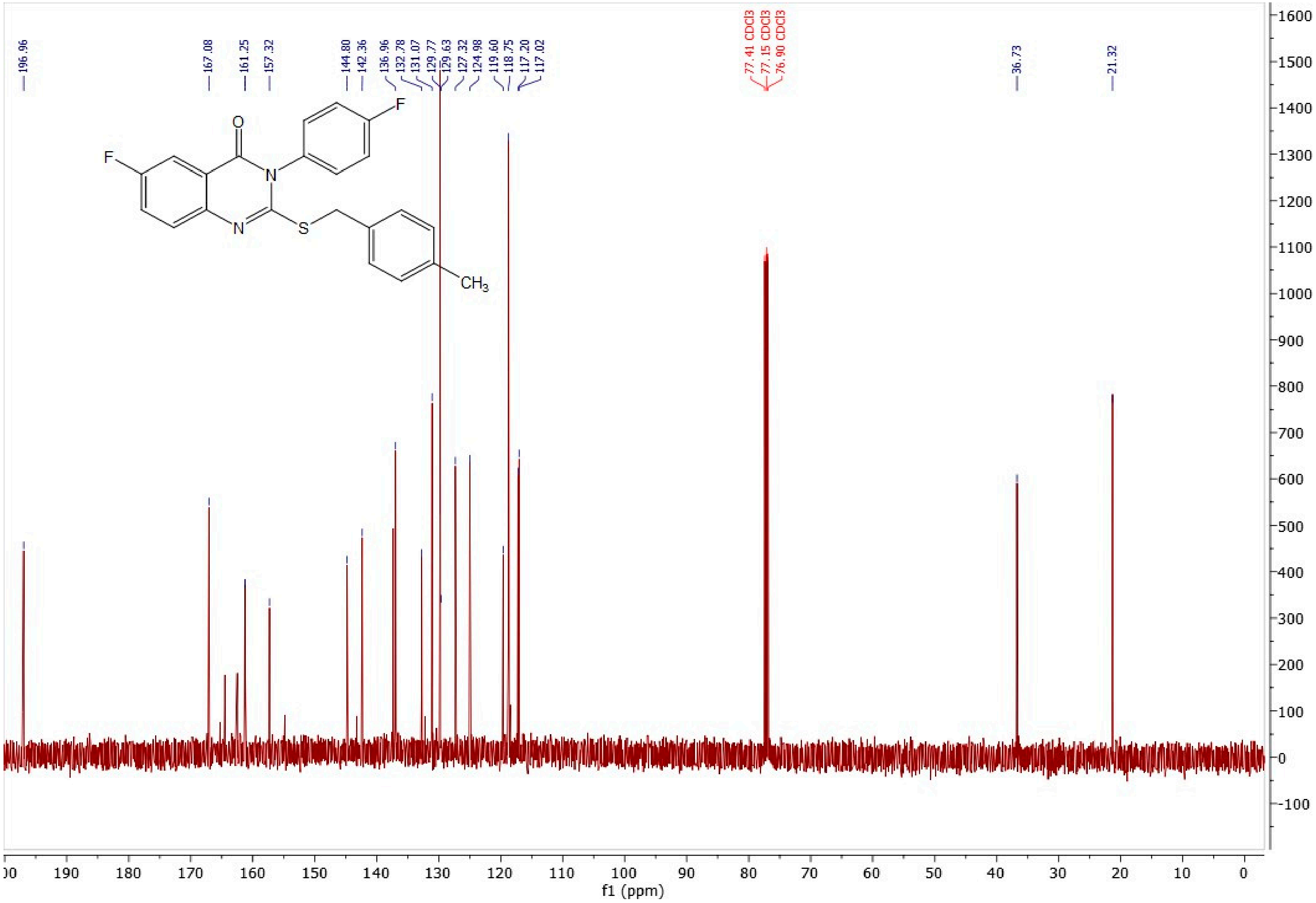

Figure S4. ^{13}C NMR of intermediate **5a**.

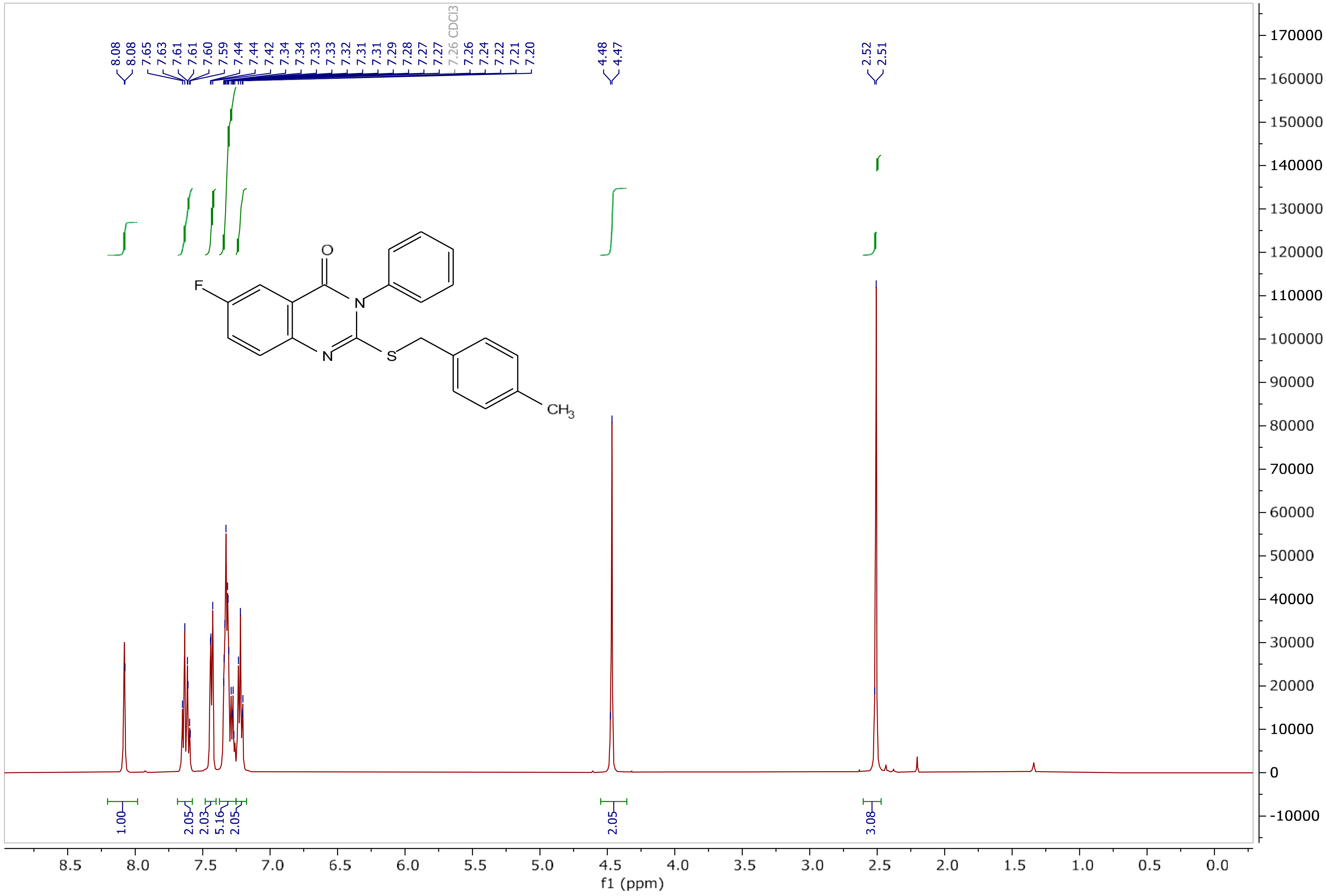

Figure S5. ^{1}H NMR of intermediate **5b**.

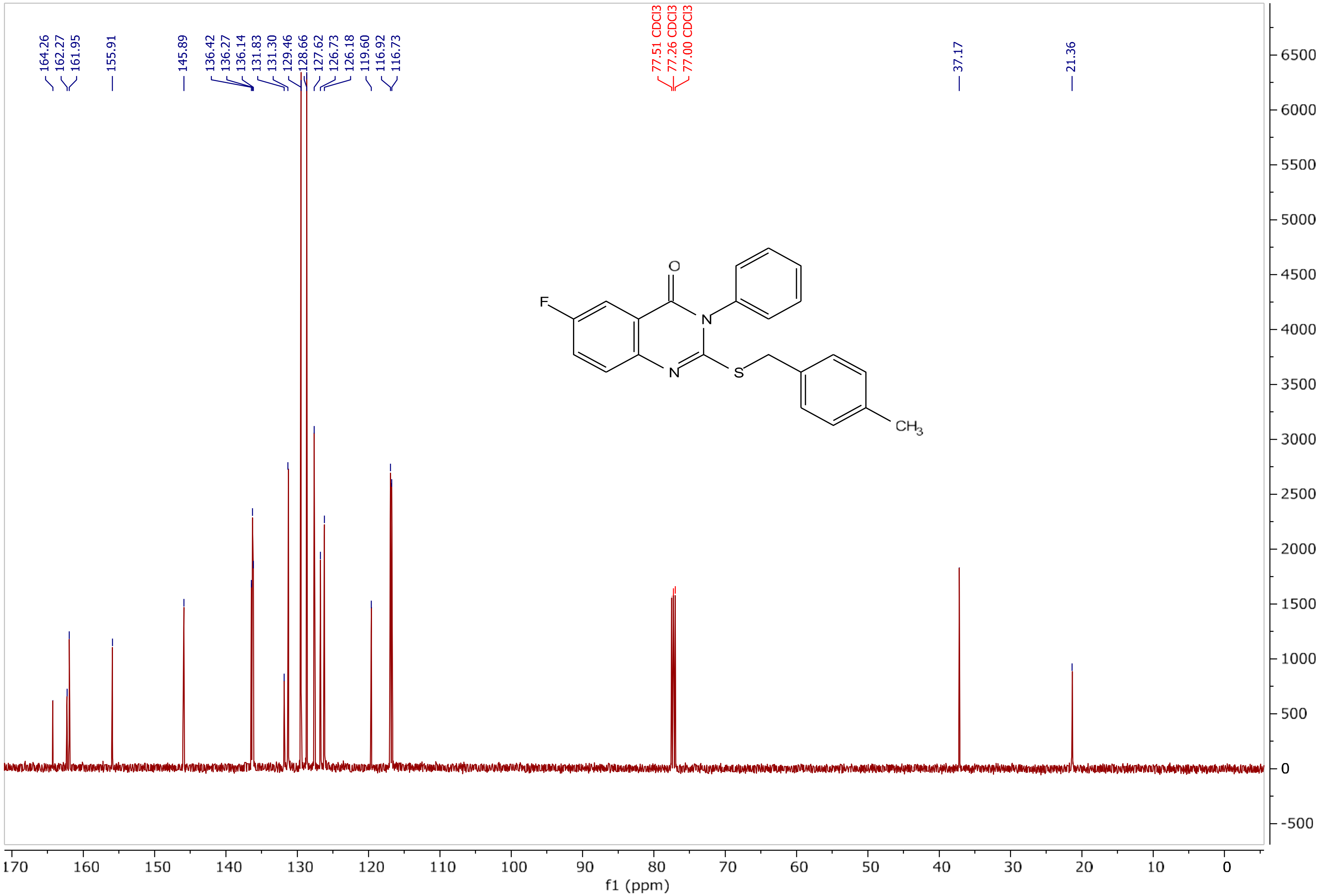

Figure S6. ^{13}C NMR of intermediate **5b**.

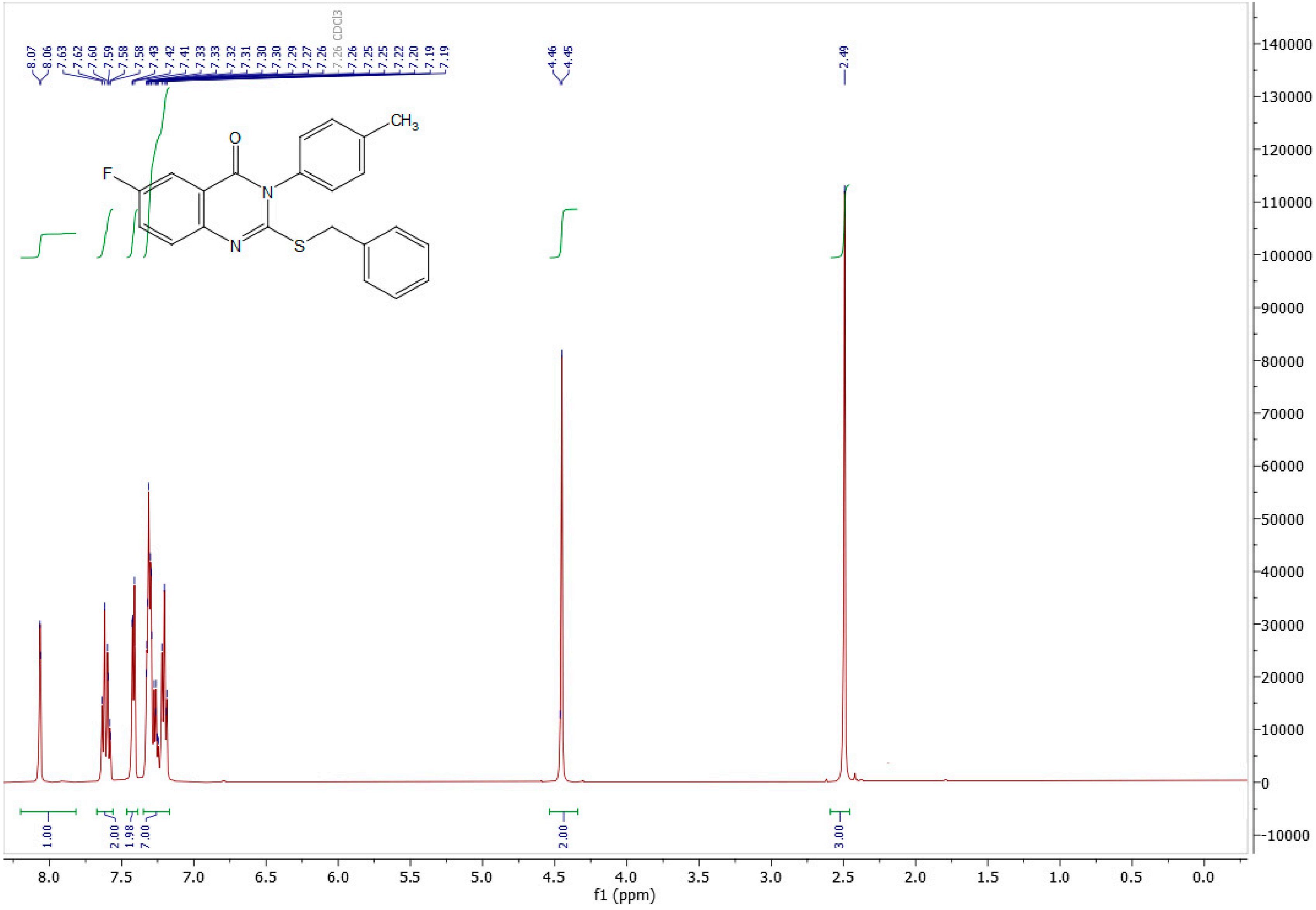

Figure S7. ¹H NMR of intermediate **5c**.

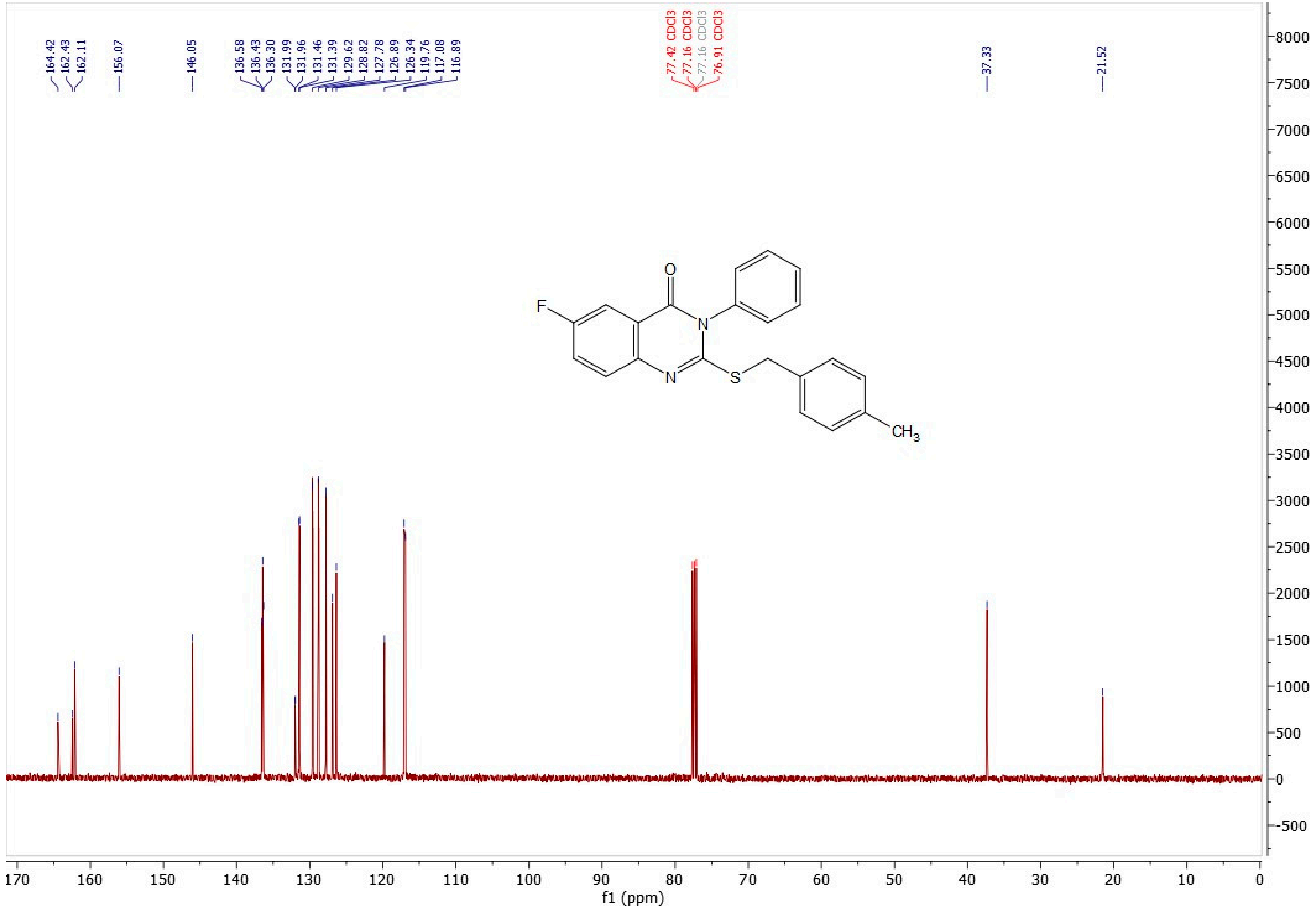

Figure S8. ^{13}C NMR of intermediate **5c**.

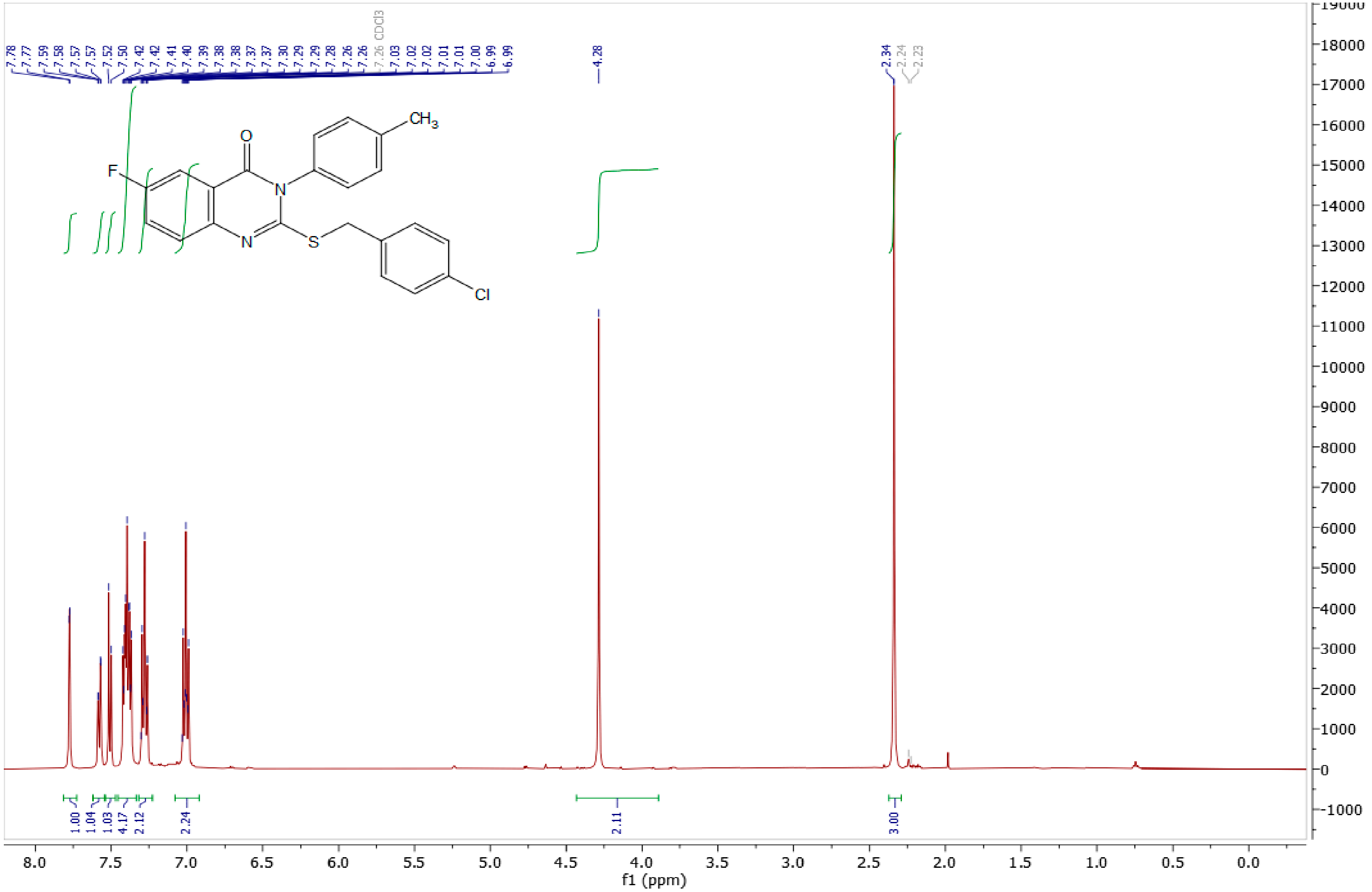

Figure S9. ^{1}H NMR of intermediate **5d**.

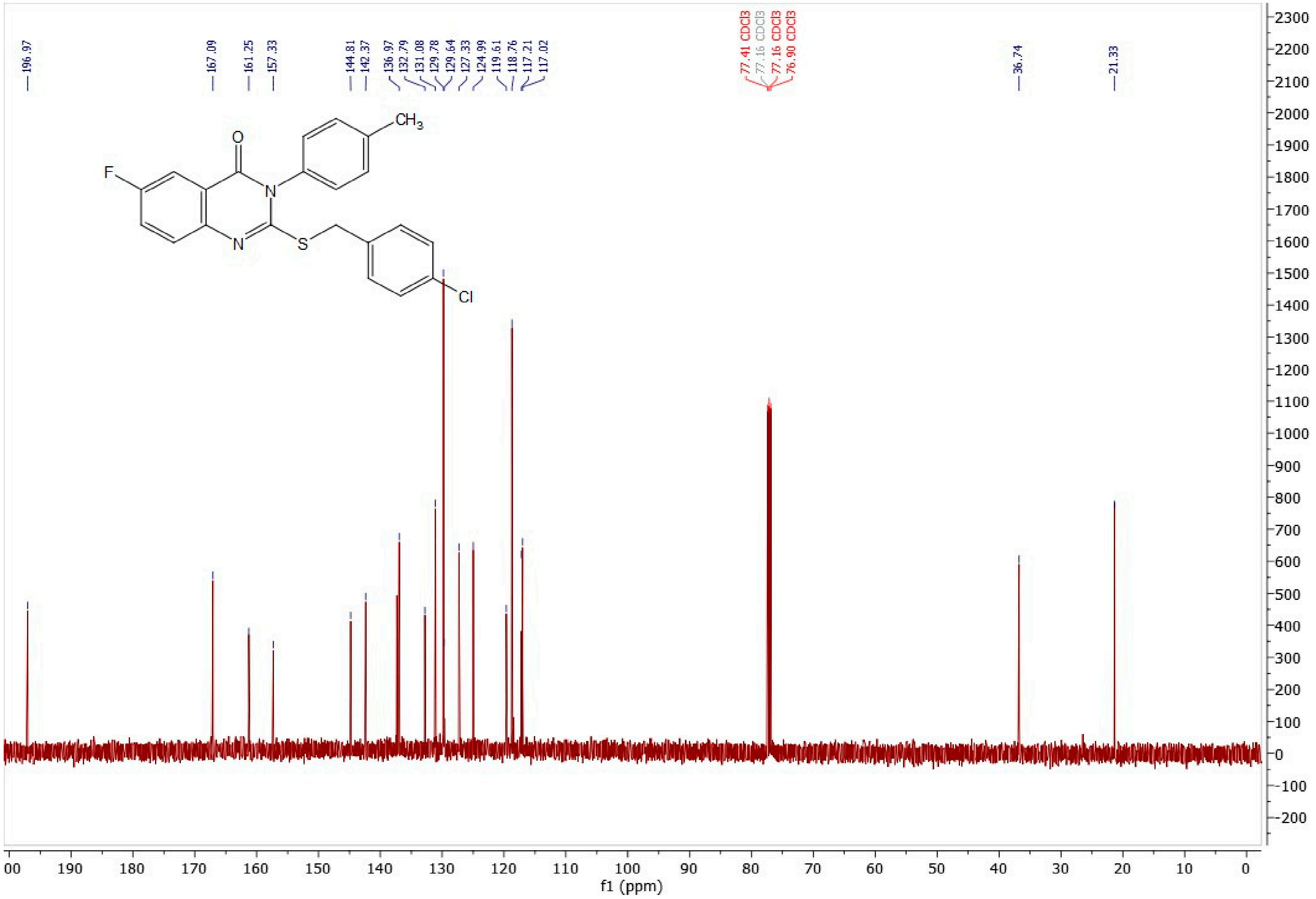

Figure S10. ^{13}C NMR of intermediate **5d**.

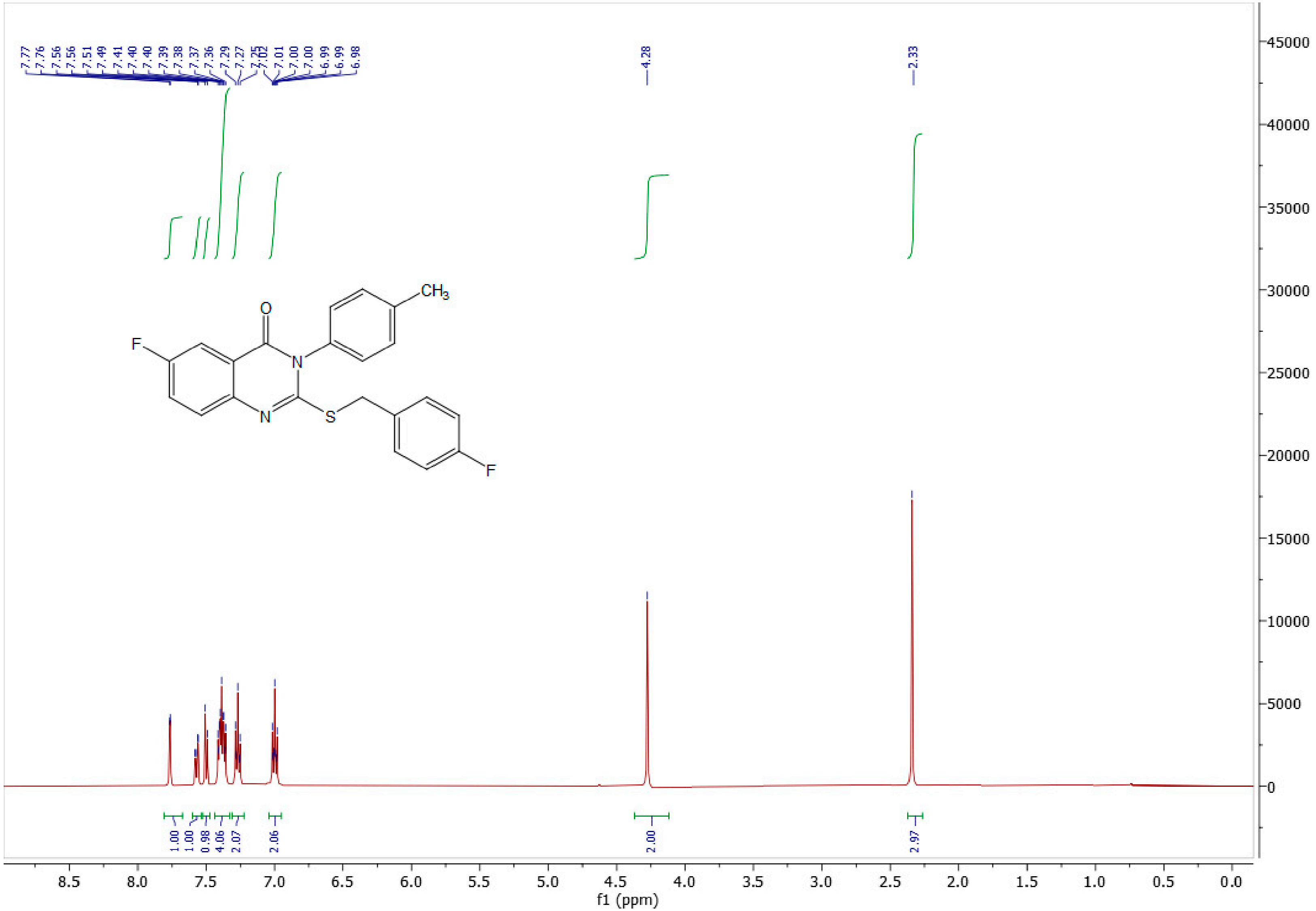

Figure S11. ^{1}H NMR of intermediate **5e**.

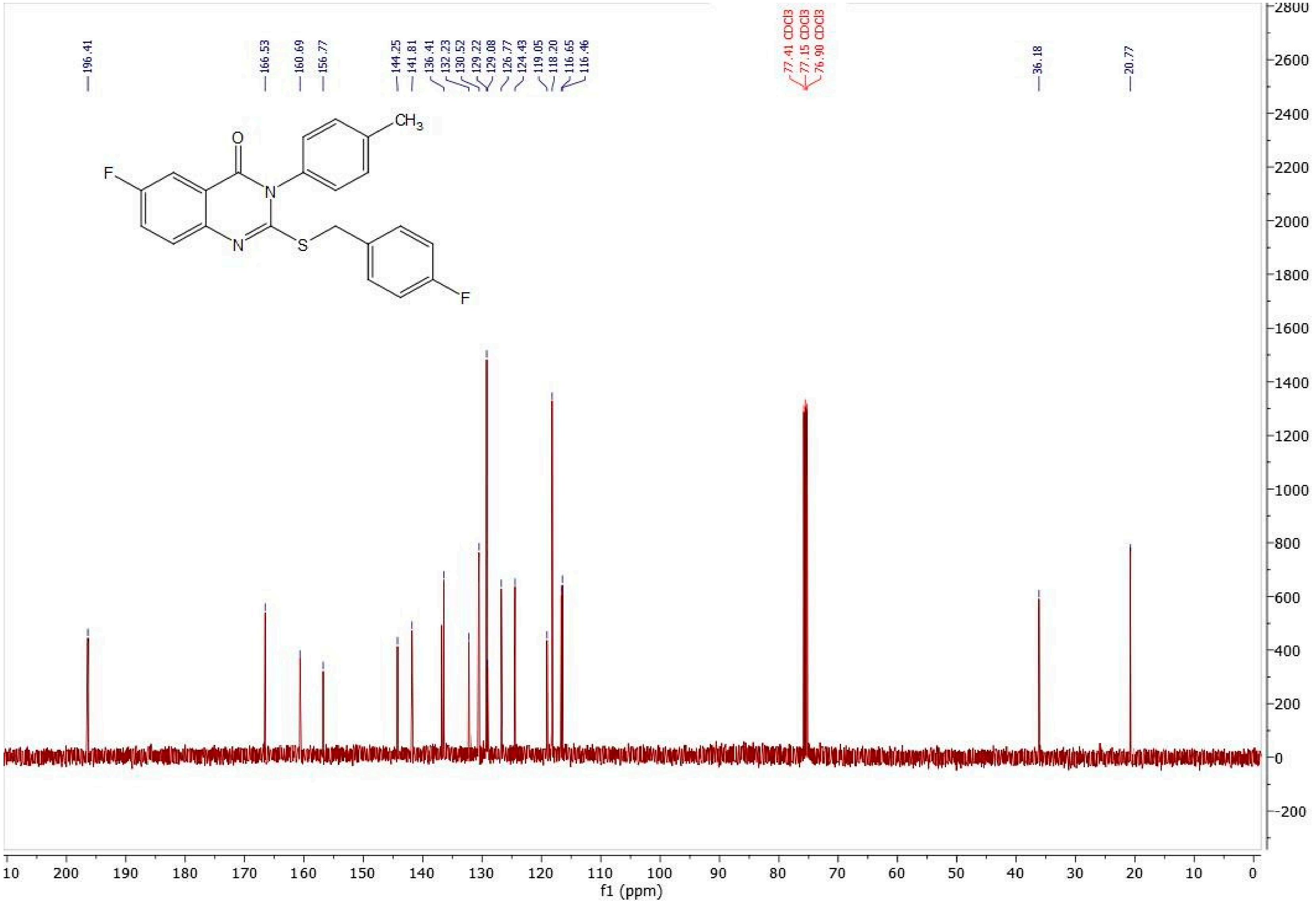

Figure S12. ^{13}C NMR of intermediate **5e**
